# Scaly rash and a mite: If not scabies, why scabies shaped?

**DOI:** 10.1016/j.jdcr.2026.03.010

**Published:** 2026-03-13

**Authors:** Jonathan D. Ho, Chelsea Roye

**Affiliations:** a Division of Dermatology, Department of Medicine, The University of the West Indies, Mona Campus, Kingston, Jamaica; b Department of Pathology, The University of the West Indies, Mona Campus, Kingston, Jamaica

**Keywords:** dermatophagoides, dust mite, environmental mite, misdiagnosis, sarcoptes scabiei, scabies, skin of color

## Case presentation

**Fig 1:**
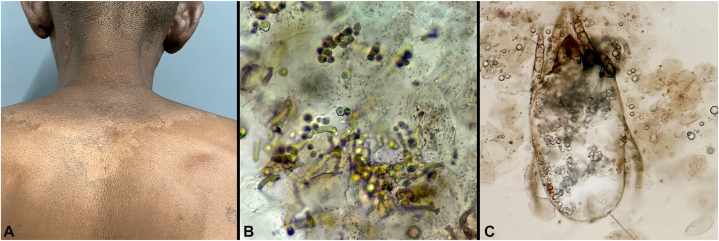
Thin scaly plaque on upper back **(A)**. Skin scraping (chlorazol black), reveals fungal yeast and hyphae in a spaghetti and meatball arrangement **(B)**. Additionally, a well preserved mite was identified **(C)**.

A 42-year-old male presented for evaluation of a mildly pruritic rash to the upper back, present for over 1 year. He had no chronic medical illnesses, took no medications and had no history of drug allergies. Physical examination revealed a thin scaly plaque on his upper back (Fig 1, *A*). Similar lesions were present on the anterior trunk. Skin scraping with chlorazol black stain was performed and revealed fungal hyphae and yeast with a “spaghetti and meatball appearance” (Fig 1, *B*) and a mite (Fig 1, *C*).

**Question: How would you manage this patient?**

**A.** 2% ketoconazole cream only
**B.** 2% ketoconazole cream and 5% permethrin cream
**C.** 2% ketoconazole cream and oral ivermectin
**D.** Fluconazole and oral ivermectin
**E.** 2% ketoconazole cream, oral ivermectin, and HIV serology

## Discussion

The correct answer is **A**.

This patient has tinea versicolor and a dust mite. Environmental mites may be incidentally seen in or contaminate skin scrapings. Misidentification may lead to inappropriate treatment. Dust mites (*Dermatophagoides sp*) in particular, are free living, common environmental mites and may be carried on clothing, or be present on dusty glass slides.^1^ They are, superficially, morphologically similar to, *Sarcoptes scabiei*. Although comfortable identifying other mites causing human disease (eg, *Demodex sp.,*), dermatologists may be less familiar with dust mite morphology. While detailed analysis requiring high quality microscopy, mite preservation, and parasitology expertise is required for exact speciation (there are numerous species),^2^ recognizing simple features allow quick differentiation, avoiding a diagnostic pitfall.

**Fig 2:**
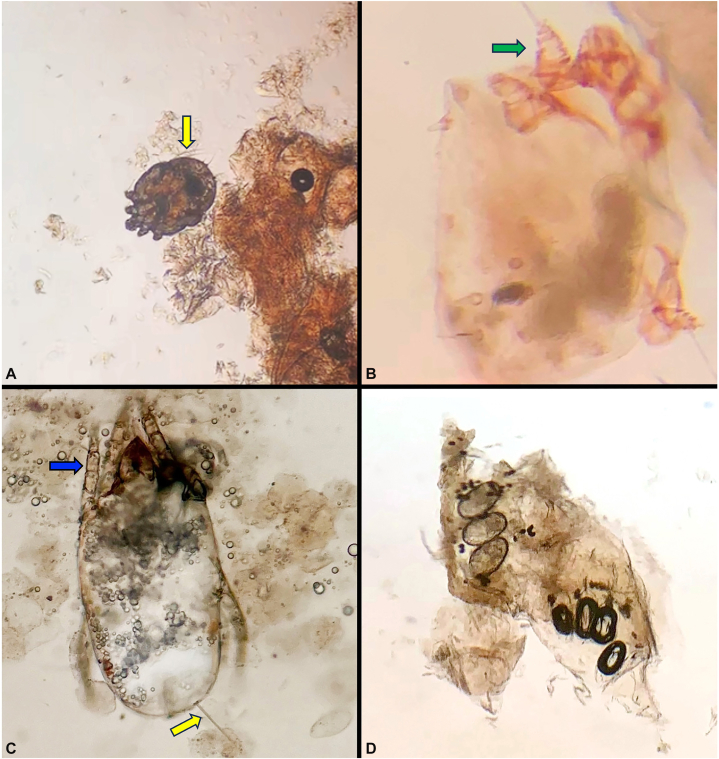
Scabietic and dust mites. Compared with dust mites, scabietic mites are rounder, appear flattened and have a tortoise-like appearance **(A** and **B)** with short stump-like legs **(A)**. The anterior legs are more easily visible from the dorsal view **(A)**. In addition to being short, scabietic mite legs are distinctly triangular/cone shaped **(B,***green arrow*). In contrast, dust mites **(C)** are often more elongate and (along with many other free-living mites) have longer legs with easily visible segments **(C,***blue arrow*). Both mites have bristles (setae, *yellow arrows*). Although both produce similar eggs and feces, as dust mites are only incidental/transient in skin scrapings, the presence of either favors scabies **(D)**.

Evaluation of the body shape and leg length and shape can quickly distinguish environmental contaminant/incidental mites from scabies. Both mites have 8 legs, a visible mouth piece and bristles (setae, Fig 2, yellow arrows).^2^ Scabietic mites (Fig 2, *A* and *B*) have a round, flat, tortoise-like shape, while dust mites are elongate and less flat (Fig 2, *C*).^3^^,^^4^ Scabietic mites have short stump-like legs (Fig 1, *A*). The anterior legs are more easily seen unless the ventral aspect of the mite is appreciated. Dust mites (and many other environmental mites) have longer, thinner legs (Fig 1, *C*, blue arrow). The segmented/jointed nature is easily appreciated. Scabietic mites have distinct triangular/cone shaped legs, a feature absent in dust mites (Fig 2, *B*, green arrow).^4^^,^^5^ Additionally (but less easily seen), scabietic mites have a parallel patterning on their cuticle giving a finger print-like appearance.^4^^,^^5^ Both sets of mites have identifiable eggs and feces, but since dust mites are only incidentally/transiently present on human skin scrapings, the presence of eggs and feces is unlikely and favor scabies (2D).Taken together, these features help dermatologists to avoid misclassifying contaminant/incidental mites as *S scabiei.*

## Conflicts of interest

None disclosed.
